# The long crisis of Italy’s National Health Service: universalism on paper, inequity in practice

**DOI:** 10.1093/eurpub/ckag138

**Published:** 2026-07-30

**Authors:** Nicola Abrescia, Adelaide Maddaloni, Gabriella Molinaro, Antonio Capponi, Maurizio D’Abbraccio

**Affiliations:** AORN Dei Colli, D. Cotugno Hospital for Infectious Diseases, Naples, Italy; AORN Dei Colli, D. Cotugno Hospital for Infectious Diseases, Naples, Italy; Faculty of Medicine and Surgery, School of Nephrology, University of Naples Federico II, Naples, Italy; Faculty of Veterinary Medicine, School of Infectious Diseases, University of Naples Federico II, Naples, Italy; AORN Dei Colli, D. Cotugno Hospital for Infectious Diseases, Naples, Italy

Italy’s Servizio Sanitario Nazionale (SSN), established in 1978, remains one of Europe’s most recognizable tax-funded universal health systems [1]. Its universalist commitment has not formally been abandoned. The more serious problem is that universalism has become increasingly nominal: patients may retain legal entitlement to care while being forced to wait, travel considerable distances, or pay privately to obtain it. The crisis is therefore not the disappearance of rights, but the weakening of the public system’s capacity to make those rights effective.

This is not a sudden crisis of the SSN, but a long erosion of operational access within a system that retains a formally universal architecture. The recent suspension of Italy’s proposed reform of general practice did not cause this erosion. Its relevance lies elsewhere: it exposes the unresolved governance of territorial care, the level at which formal universalism is converted, or fails to be converted, into usable access [2]. For European public health, the Italian case raises a wider question: what remains of universal health care when financing, workforce, territorial services, and regional governance no longer sustain timely and equitable access?

Italy’s aggregate health indicators remain comparatively favourable. The country continues to report relatively low avoidable mortality and comparatively low admission rates for selected chronic conditions [3]. Such indicators, however, may coexist with a deteriorating experience of access, and therein lies the analytical importance of the Italian case. It demonstrates how universal systems may erode without formally abandoning universal coverage. The mechanisms are interdependent, but they can be examined through five operational axes. Time identifies rationing through delay; capacity identifies the mismatch between demand and system capability; spatial organization captures the tension between efficiency, safety, and proximity; regional inequality shows how decentralized delivery can produce unequal citizenship; and territorial reform exposes the gap between policy architecture and operational access. Together, these axes explain how universal entitlement can persist while effective access becomes increasingly unequal.

Time is the first and most visible axis. Waiting lists are not simply an administrative inconvenience; they function as a rationing mechanism through which legal entitlement becomes delayed or foregone care. In 2024, 9.9% of the Italian population reported forgoing visits or clinical tests for economic, organizational, or service-availability reasons, with waiting lists the leading cause, cited by 6.8% of the population, followed by difficulty paying for services, at 5.3% [4]. These figures capture foregone care rather than disease-specific harm, yet their policy significance is clear: across cancer diagnosis, cardiovascular assessment, imaging, surgery, rehabilitation, and chronic-disease follow-up, delay can transform a universal right into either a clinically consequential wait or a privately purchased shortcut.

Capacity is the second axis. In 2023, Italy’s health expenditure stood at 8.4% of GDP, below the EU average, with public sources accounting for 73% of total health spending, again below the EU average [3]. Underfunding alone does not explain inequity, but it compounds workforce shortages, ageing infrastructure, weak long-term care provision, fragmented organization, and rising out-of-pocket expenditure. The limiting factor is not the competence of Italian professionals, but the capacity of the system to organize sufficient staff, services, and care pathways around clinical need.

Spatial organization is the third axis. Hospital planning has increasingly relied on volumes, bed occupancy, tariffs, diagnosis-related group classifications, and network standards [5]. These criteria have a legitimate safety and efficiency rationale: centralization can improve outcomes when volumes and expertise matter, and not every local service can or should be preserved unchanged. The equity problem arises when centralization proceeds without adequate attention to geography, transport time, emergency capacity, and credible territorial alternatives. In that setting, rationalization may produce distance-related inequity, particularly for rural and peripheral populations. A universal health service must distinguish inefficient duplication from necessary clinical presence.

Regional inequality is the fourth axis. The SSN guarantees national entitlements, but delivery depends upon regional health systems with unequal fiscal capacity, infrastructure, workforce strength, and political priorities. Regional autonomy can allow local adaptation, but it can also institutionalize unequal access when national standards are weakly enforced. Interregional health mobility is therefore more than a marker of inequality; it can amplify it. AGENAS has documented persistent hospital and outpatient flows across regional boundaries; GIMBE, drawing on interregional compensation data, estimated the aggregate value of such mobility at €5.153 billion in 2023 [6, 7]. When patients, resources, and professionals move systematically from less-resourced, often southern regions towards better-resourced, predominantly northern regional systems, mobility ceases to be a freedom and becomes an expression of unequal citizenship.

Unfinished territorial reform is the fifth axis. Under DM77/2022, Case della Comunità are designed as community health hubs integrating primary care, nursing, social-health services, home-care coordination, basic specialist pathways, and hospital continuity [8]. Yet by the end of 2025, AGENAS reported that 781 of 1715 planned sites had at least one active service, with only 66 offering all mandatory services required under DM77 standards [9]. This gap matters because hospital rationalization can be equitable only if credible territorial alternatives exist. Organizational diagrams and construction targets do not create proximity care unless staffed services, diagnostics, nursing, social support, digital continuity, and accountability for access are operationally present.

Private provision also requires a proportionate interpretation. In some settings it may absorb demand, offer flexibility, and shorten queues for those who use it. The equity question is not whether private provision, regional autonomy, or centralization are illegitimate in principle; it is whether they are governed so that timely access does not depend on residence, income, or local administrative capacity. Without such governance, these mechanisms do not merely coexist with universalism; they can hollow it out while leaving its legal form intact.

General practice is the operational hinge of territorial access. The core issue is functional integration: general practice must be embedded in multidisciplinary teams, diagnostics, home-care pathways, chronic-disease management, and community health structures so that proximity care becomes operational rather than merely nominal. Italian general practitioners are self-employed physicians contracted through a convention-based model rather than salaried SSN employees [10]. This contractual status is not, in itself, the obstacle to equitable access, but it shapes how readily integration, shared accountability, and territorial obligations can be organized. Home visits are formally governed by the criterion of patient non-transferability, which is necessary for workload management but does not always capture serious acute illness, progressive frailty, rapid deterioration, or social isolation. Remote consultation may improve convenience, but when used principally as a workload filter it risks deepening inequity for patients who are frail, digitally excluded, or clinically complex. General practice reform therefore matters because it determines whether territorial care becomes an operational access platform or remains a fragmented gatekeeping layer.

Italy’s long crisis should not be misread as evidence that universalism has failed as a principle. It is evidence of a failure to align universal entitlement with measurable, enforceable access. The policy implication is concrete but not prescriptive. Universal systems require waiting-time governance by priority and service line; routine monitoring of unmet need and foregone care; territorial service standards calibrated to geography, travel time, and clinical risk; workforce distribution anchored in demographic need; integration of primary care with diagnostics, home care, and community services; and regional accountability for access gaps. These domains should be evaluated not by infrastructure announcements or legislative ambition, but by the services actually available to patients.

For Italy and for Europe, the test of universalism is no longer only whether rights are enumerated in law. It is whether patients can obtain care in time, near enough, and without paying privately to bypass public delay. When that test fails, universalism persists on paper while inequity governs practice.

Key points

- Italy’s SSN remains formally universal, but effective access is progressively undermined by waiting times, geography, regional capacity disparities, workforce scarcity, and growing out-of-pocket expenditure.
- Five operational axes account for this erosion: time, systemic capacity, spatial organization of services, regional inequality, and unfinished territorial reform.
- Centralization, regional autonomy, and private provision are not inherently inequitable, but they require governance to prevent access from depending on residence, income, or local administrative capacity.
- Territorial reform can restore equity only if community hubs, general practice, home care, diagnostics, and workforce planning are operationally integrated.
- European universal health systems should be evaluated by the effective access they deliver, not solely by the legal entitlements they confer.

## Data Availability

No original data were generated for this article. All sources cited are publicly available.
